# Action of Chloral Hydrate

**Published:** 1873-11

**Authors:** Wm. Kirkpatrick Murphy


Action of Chloral Hydrate.—*Wm. Kirkpatrick Murphy, M.D.* Whilst disposed to grant the incalculable benefits to be derived from hydrate of chloral as an occasional or temporary hypnotic or sedative in acute disease, and where not contra-indicated, I think sufficient evidence has been adduced, not merely to render the policy of its employment in chronic affections very questionable, but to suggest the total impropriety of its administration in chronic disease at all. To be sure, it may be objected that chloral is comparatively a new medicine, and cannot yet have been thoroughly tested; and with equal fairness it may be asserted, that its value as a therapeutic agent is not to be appraised by the *ipse dixit* of a solitary practitioner. Both propositions are indisputably just. To both, however, I would with deference submit in reply the data recorded in the above cases. They constitute a series of reliable clinical facts, personally observed and attested, and embrace within themselves, it seems to me, all the elements necessary to the deduction of a fair and legitimate inference. They may be said to be typical cases, in fact, presenting such characteristic variations in detail as to render their approximate uniformity in result altogether remarkable. Thus the original malady in each case was different, as also were the constitutions of each, and their susceptibility to hypnotic influences. Similarly, too, the quantity of chloral exhibited, the times and modes of its administration, and the duration of its continued use—six, eighteen, and twenty-four months, respectively—and last, but not least, the authority and supervision, whether those of a professional attendant or simply, as in Case 3, the patient's own, under which it began and continued, all present diversities sufficiently marked and peculiar. Compare, then, these diversities in malady, constitution, and treatment, with the curious similarity manifested in the individual symptoms of each, and with the absolute identity presented by all in general tone and tendency, and what follows? The conviction, I think, must be forced upon every candid mind, that the protracted use of chloral, especially in its application to chronic disease, is equally impolitic and unjustifiable, inasmuch as it has been shown, I would hope conclusively, that when long administered it is not less insidious in its nature that it is unquestionably pernicious in its effects.

